# Investigating the Effect of Blurring and Focusing Current in Cochlear Implant Users with the Panoramic ECAP Method

**DOI:** 10.1007/s10162-024-00966-x

**Published:** 2024-10-16

**Authors:** Charlotte Garcia, Charlotte Morse-Fortier, François Guérit, Scott Hislop, Tobias Goehring, Robert P. Carlyon, Julie G. Arenberg

**Affiliations:** 1 Cambridge Hearing Group, MRC Cognition & Brain Sciences Unit, University of Cambridge, 15 Chaucer Road, Cambridge, CB27EF UK; 2 Eaton Peabody Laboratories (EPL), Massachusetts Eye and Ear, Harvard Medical School, 243 Charles Street, Boston, MA 02114 USA

**Keywords:** Cochlear implant, Electrically evoked compound action potential (ECAP), Focused stimulation, Blurred stimulation, Current spread, Neural responsiveness

## Abstract

**Purpose:**

For some cochlear implants (CIs), it is possible to focus electrical stimulation by partially returning current from the active electrode to nearby, intra-cochlear electrodes (partial tripolar (pTP) stimulation). Another method achieves the opposite: “blurring” by stimulating multiple electrodes simultaneously. The Panoramic ECAP (PECAP) method provides a platform to investigate their effects in detail by measuring electrically evoked compound action potentials and estimating current spread and neural responsiveness along the length of the CI electrode array. We investigate how sharpening and broadening the electrical current spread are reflected in PECAP estimates.

**Methods:**

PECAP measurements were recorded at most comfortable level in 12 ears of Advanced Bionics CI users. Focused thresholds were also determined. For the electrodes with the highest and lowest focused thresholds, additional PECAP measurements were recorded while stimulating in pTP mode and in “blurred” mode with 3 or 5 adjacent electrodes simultaneously stimulated. Current spread and neural responsiveness were then estimated along the electrode array using PECAP.

**Results:**

PECAP revealed increased current spread estimates across participants for blurred stimulation of the targeted electrodes towards the apex of the cochlea. Variable results for pTP stimulation were found, with two of eight ears appearing to drive a small group-level effect of increased current spread.

**Conclusion:**

When stimulating multiple electrodes simultaneously, PECAP detected localized increases in current spread towards the apex (but not the base) of the cochlea. pTP stimulation showed mixed effects on PECAP current spread estimates. These findings are in line with behavioral speech perception studies and have implications for cochlear implant optimization.

## Introduction

Although many cochlear implant (CI) users obtain good speech perception with their implant in quiet backgrounds, many still struggle to understand speech, especially in challenging listening environments, such as those that contain background noise [1, 2]. One of the limitations of CIs is the reduced spectral resolution that is delivered to the auditory nerve through the implant compared to acoustic hearing [3]. While an acoustically stimulated cochlea can encode the frequency spectrum using thousands of hair cells organized tonotopically along the length of the basilar membrane, a CI replaces this functionality with only between 12 and 22 intra-cochlear electrodes. This diminished number of distinct frequency channels can be further reduced by poor electrode-neuron interfaces and channel interaction effects, wherein the populations of auditory neurons that are stimulated by one electrode are also stimulated by other electrodes [3–5]. This reduces the efficiency of delivering auditory information through the CI to the auditory nerve and onward to the language-processing networks in the brain.

There have been multiple attempts to focus current in CI users and improve spectral selectivity by adjusting the method of stimulation with varying degrees of success [3, 6, 7]. The present study investigated one such method for attempting to focus the spread of electrical current, and a second method that instead attempts to *broaden* the spread of electrical current. Both benefit from CI platforms that contain multiple current sources for simultaneous stimulation at multiple electrodes.

The focusing method involves tripolar (TP) stimulation wherein active current is delivered to a central electrode and the current is returned via the two adjacent electrodes. This modality was originally shown to focus electrical stimulation mode compared to monopolar (MP) mode (wherein the current is returned to an electrode outside of the cochlea) in cat cadaver and tank experiments, as well as in living cats [8] and Guinea pigs [9, 10]. In many cases, in order to achieve the required perceptual loudness levels in humans, a fraction of the current is also returned to extra-cochlear electrodes as in MP mode, and we refer to this as partial tripolar (pTP) stimulation [11, 12]. It has been shown that stimulating in partial tripolar mode may produce narrower excitation profiles than when stimulating in the standard monopolar mode [12, 13]. Results for improving perception using these focused stimulation approaches are mixed: various studies have shown improvement in speech perception at the group level [14], virtual channel discrimination [15], and polyphonic pitch perception [16]. However, other studies have not shown any improvement in speech perception or spectral selectivity when employing focused stimulation [17–19].

The broadening method for manipulating current spread involves stimulating multiple adjacent electrodes simultaneously and in the same polarity, which is expected to *degrade* spectral resolution and has been shown to impair speech perception [20–22]. These studies used a method called “blurring” by attempting to *blur* spectral information and increase channel interaction by simultaneously stimulating multiple electrodes. They found that performance on speech-in-quiet and speech-in-noise tests was degraded when all channels were blurred (where a “channel” refers to the central electrode when multiple are simultaneously stimulated), and that CI users’ susceptibility to this effect was associated with their performance on a spectro-temporal test [20, 21]. Goehring et al*.* further found that blurring a set of 5 electrodes at the apical end of the electrode array affected speech scores, but that blurring 5-electrode sets in the middle or basal part of the array did not [22]. They speculated that this selective effect of blurring may be due to the greater importance of spectral information to speech intelligibility in the lower frequencies relative to the spectral information at higher frequencies, in line with other studies’ findings in CI listeners [23, 24]. However, there may also be physiological or device-related reasons for a location-specific effect of blurring along the cochlea. The evidence to date does suggest that a wider current profile or more channel interaction is achieved when stimulating with multiple electrodes at once than when stimulating in MP mode with a single electrode or in sequential mode [25]. However, there is variation between CI users as to how many electrodes are needed to be simultaneously stimulated in order to degrade spectral resolution and/or speech perception. The underlying current profiles involved in this method of “spectral blurring” are also not understood in detail. Blurring is therefore a useful way to validate the sensitivity of methods designed to measure the spread of current and neural excitation in CI users.

To assess differences in neural excitation profiles as a result of stimulating with the methods described above, this study used electrically evoked compound action potentials (ECAPs). ECAPs are a measure of the synchronized response of the auditory nerve fibers to stimulation with a current pulse from a CI. They can be recorded by sending an electrical current pulse through one electrode in the implant and using a nearby electrode to measure the resulting voltage profile of all responding neurons. The Panoramic ECAP (PECAP) method is a method of recording multiple ECAPs and using an optimization model to extract estimates of current spread and neural responsiveness along the length of the CI electrode array [26, 27]. The accuracy of the neural responsiveness estimate has previously been validated using localized regions of reduced neural responsiveness, simulated by presenting pre-pulses on one chosen “dead” electrode to put nearby neurons into a refractory state prior to recording ECAPs. Garcia et al*.* show that the PECAP algorithm correctly attributes this manipulation to a reduction in the neural responsiveness estimate surrounding the chosen “dead” electrode and not to the remainder of the neural responsiveness estimate nor to the current spread estimate [26]. However, PECAP’s estimate of current spread has only previously been validated using somewhat circular computer simulations.

The purpose of this study is twofold: First, we wanted to investigate whether PECAP could correctly attribute a manipulation to current spread such as blurring or focusing with pTP stimulation to a change in the targeted electrode’s current spread estimate, and not to a change in the neural responsiveness estimate. If found, this effect would provide evidence in favor of PECAP’s ability to accurately estimate current spread in CI users and in turn confirm that the blurring or focusing method can effectively manipulate current spread and resulting neural excitation patterns.

Second, we wanted to understand the effect of blurring and partial tripolar stimulation on neural excitation profiles in greater detail. The PECAP method also provides a platform to investigate potential causes of variations between implant users and different electrodes. It was hypothesized that if current spread was already relatively wide in MP mode, then blurring electrical current would have a smaller effect compared to when the current spread was relatively narrow in MP mode. It was also hypothesized that if the pTP stimulation is near an area of relatively poor neural health, it might be less effective at focusing the current than if it is employed near an area of relatively healthy neurons. To localize areas of relatively poor vs good neural health, we used focused thresholds as these have been shown to correlate with other measures understood to reflect neural health [26, 28]. Focused thresholds have also been shown to predict areas of poor spatial selectivity, wherein wider psychophysical tuning curves have been observed for electrodes that show high focused thresholds than when compared to electrodes with low focused thresholds in the same participants [29]. It may therefore be that the effects of manipulating current spread could differ between electrodes with high or low focused thresholds.

## Materials and Methods

### Participants

Thirteen (13) adult users of Advanced Bionics CIs were recruited, two of whom were bilaterally implanted and completed the study with both ears for a total of fifteen (15) ears. Demographic information can be found in Table 1, in addition to some experimental details. Inclusion criteria required that it was possible to collect PECAP data that achieved a 10 decibel (dB) or higher signal-to-noise ratio (SNR), as specified for achieving accurate neural excitation profiles with PECAP [26]. Three (3) ears did not achieve this threshold (AB011R, AB018R, and AB020L, indicated by the asterisks * in Table 1) and were excluded from analysis, leaving a total of twelve (12) ears in the primary dataset. Further details for calculating the SNR of the PECAP matrices and other study-specific inclusion criteria for the different parts of the analysis are described below in the “Electrically Evoked Compound Action Potentials” section of the “Materials and Methods” section.

**Table 1:** Participant demographic information (*R* right, *L* left, *yrs* years, *Elecs* electrodes, *pTP* partial tripolar; * indicates that the participant was excluded from analysis due to low PECAP SNR values; ^F^electrode where pTP stimulation was applied, ^B^electrode where blurred stimulation was applied, **^**electrode that showed high sQP thresholds)

ID	Ear	Electrode array type	Age (yrs)	Duration of profound hearing loss before implantation (yrs)	Duration of implant use (yrs)	Aetiology	PECAP details				
							Level rating	Elecs	SNR (dB)	Blurred and/or focused Elecs	*α* coefficient for pTP stimulation (apical/basal)
										*(****^bold**** indicates high sQP)*	
AB001	L	SlimJ	31	28	3	Congenital, genetic	7	1–16	19.9	6^B,F^, **^12**^B,F^	0.9/^**0.9**
AB002	L	Helix	61	11	17	Sudden hearing loss	6	1–16	19.6	**^8**^B,F^, 12^F^	**^0.6**/0.5
	R	Helix		12	15	Progressive sensorineural	6	1–16	23.5	5^B^, **^9**^B^	n/a
AB005	L	Helix	76	9	12	Progressive sensorineural	8	1–16	10.9	6^B,F^, **^10**^B,F^	0.7/^**0.7**
AB007	R	SlimJ	60	2	3	Meniere’s	6	1–16	16.3	**^5**^B,F^, 10^B,F^	**^1.0**/0.5
AB008	R	Mid-Scala	25	9	1	Unknown	8	1–15	16.1	6^B,F^, **^10**^B,F^	0.6/^**0.5**
AB011	L	1 J	59	11	18	Progressive sensorineural, genetic	6	1–16	15.0	5^B,F^, **^12**^B^, **^13**^F^	0.6/^**0.6**
	R	Helix		15	15		6	1–16	4.96*	**^5**^B,F^,8^F^	**^0.6**/0.6
AB013	L	Mid-Scala	32	26	3	Genetic	7	1–15	10.8	7^B,F^, **^11**^B^, **^12**^F^	0.6/^**0.7**
AB014	L	Mid-Scala	67	3	6	Meniere’s	6	1–16	19.5	5^B^, **^11**^B^	n/a
AB015	L	Mid-Scala	50	1	8	Meniere’s	7	1–15	16.7	**^5**^B,F^, 11^B,F^	**^0.8**/0.8
AB016	L	SlimJ	41	1	4	Progressive sensorineural	6	1–15	16.1	**^6**^B^, 10^B^	n/a
AB017	L	Mid-Scala	45	1	7	Unknown, progressive	7	1–15	10.7	5^B^, **^10**^B^	n/a
AB018	R	Mid-Scala	32	39	4	Progressive sensorineural	7	1–16	7.55*	**^8**^B^, 11^B^	n/a
AB020	L	Helix	51	0	10	Congenital	7	1–16	3.48*	6^B^, **^10**^B^	n/a

### Ethical Approval

Ethical approval for the study was obtained from the Massachusetts Eye & Ear (MEE) Internal Review Board (IRB) under Protocol # 2021P001539. All participants attended research sessions at Massachusetts Eye and Ear in Boston and provided written informed consent for their participation in the study.

### Focused Thresholds (sQP)

Steered quadrupolar (sQP) stimulation was used to determine focused thresholds along the electrode array for all ears studied. sQP thresholds have been shown to highly correlate with partial tripolar (pTP) thresholds [30], and have previously been hypothesized to reflect some aspects of cochlear neural health [31]. sQP stimulation leverages four adjacent intra-cochlear electrodes where the two middle electrodes serve as active electrodes, the two outer electrodes serve as return electrodes for a fraction of the active current, and the remainder of the current is returned to an extra-cochlear electrode. For this study, unless otherwise specified, 10% of the current was returned to the extra-cochlear electrode, and the other 90% was split evenly between the two flanking intra-cochlear electrodes. The active current is then steered between the two middle electrodes and for electrodes 3–15, is steered entirely towards the more-basal electrode. It is then steered entirely towards the apical electrode to achieve focused stimulation on electrode 2 due to physical limitations. Due to the 4-electrode configuration, focused thresholds could not be obtained at electrodes 1 and 16. The focused threshold for each targeted electrode was set as the lowest current level for which an auditory sensation was perceived.

A rapid threshold measurement procedure based on Békésy tracking was used to determine the sQP threshold for each electrode. It was implemented using custom software written in MATLAB that runs the Bionic Ear Data Collection System (BEDCS) from Advanced Bionics AG (CA, USA) in the background and carries out an adaptive forced-choice procedure in both a forward (apical to basal) and backward (basal to apical) manner. The listener pressed a button to indicate audibility while the frequency of the tone is moving (towards either the apex or base), and the stimulation level of the sQP pulse train either increased (when the button is released) or decreased (when it is pressed). Multiple sweeps in both directions were done to yield repeated observations of threshold for each electrode. Each sQP pulse train was delivered at 997.9 pulses per second (pps) for 200.4 ms (ms). For one participant, AB020L, only 80% of the current was split evenly between the two flanking intra-cochlear electrodes due to compliance limits. The procedure is described in full detail in Bierer et al*.* [30]. From the focused threshold results, two electrodes between 5 and 12 that contained both the highest and lowest focused thresholds were selected for further current manipulation in the ECAP conditions described below.

### Electrically Evoked Compound Action Potentials

#### Baseline Conditions

Electrically evoked compound action potentials (ECAPs) were recorded using the forward-masking artefact cancellation technique at comfortable loudness levels for every combination of masker and probe electrode for all active electrodes in the participants’ “MAPs” (sound processor programs) in standard monopolar mode. Amplitudes were calculated from these ECAP waveforms using custom software that identified and subtracted the first negative peak within 400 µs of the offset of the probe pulse from the following positive peak. These ECAP amplitudes constitute the ${M}_{0}$ matrix described in Garcia et al*.* that is required for the Panoramic ECAP method to provide estimates of current spread and neural responsiveness for each electrode in an individual CI [26]. Stimulation employed symmetric, cathodic-leading biphasic current pulses presented at approximately 20 pulses per second (pps) in monopolar mode with phase durations of 43.1 µs, inter-phase gaps of 0 µs, the stimulation ground electrode set to IE2 (the extra-cochlear case electrode), and equal masker and probe electrode current levels. A masker-probe interval of 600 µs was selected in order to avoid temporal overlap between stimulation artefacts and the N1 peak of the ECAP waveform. The gain of the amplifier was fixed at a value of 1000, and the amplifier sampling frequency was fixed at 56 kHz. The recording electrode for the ECAPs was set as a default to 3 electrodes apical to the probe electrode for any given ECAP recording. It was switched to 3 electrodes basal to the probe electrode when the probe was at the apical end of the array, and therefore, no electrodes were available 3 apical to the probe (this occurred when the probe was located on electrodes 1, 2, and 3). If the masker electrode was located where the recording electrode would have been by default, the recording electrode was shifted over one electrode from the default to avoid saturating the amplifier by stimulating and recording on the same electrode, and with the assumption that the effect of shifting the recording electrode by one contact would be sufficiently small to be of little relevance in comparison with the effect of the moving masker.

The loudness scaling procedure was conducted with a 10-point loudness scale using custom software written in MATLAB 2018a that leveraged the Bionic Ear Data Collection System (BEDCS) research software (versions 1.18.321 and 1.18.337) from Advanced Bionics AG (CA, USA). The custom software was controlled through a graphical user interface (GUI) used by a research audiologist wherein the stimulating current level was initialized below perceptual threshold and increased until loudness ratings of 5 (soft but comfortable), 6 (most comfortable level or MCL), 7 (loud but comfortable), and 8 (loud) were achieved. Each time an ECAP was recorded, these data and the perceptual loudness level (if indicated) were automatically saved and the ECAP waveform was plotted in real time so that the experimenter could confirm that stimulation and recording were ongoing throughout the session. This loudness scaling procedure was conducted with 10 repetitions of each ECAP recording for every 3rd electrode active in the participant’s MAP (electrodes 1, 4, 7, 10, 13, and 16). For some participants, electrode 16 was switched off and so the loudness scaling procedure was conducted on electrode 15 instead of 16 (see Table 1). The program then recorded ECAPs with the probe and the masker co-located on the same electrode for every electrode active in the participant’s MAP at the level requested by the experimenter (starting at MCL), with the non-scaled electrodes set to current levels that were interpolated on a dB scale between adjacent electrodes, and with 50 repetitions. These data are referred to as the “diagonal” of the measurement matrix. If no ECAPs were observed in the diagonal at MCL (6), the stimulus level was increased to loudness level “7” or “8.” If the experimenter then determined that the stimulation level was comfortable for the user and achieved observable ECAP waveforms, the program then continued on to record the entire PECAP ${M}_{0}$ matrix at that loudness level (again with 50 repetitions). The criteria for determining whether an ECAP was present or not was based on a comparison between the calculated ECAP amplitude and the noise floor of the recording calculated from the standard deviation of the noise-only recording frames (D, as described below), as well as visual inspection of the experimenter looking for the presence of negative and positive peaks typical of the morphology of ECAP waveforms. The loudness level used for each participant is indicated in “PECAP Level rating” in Table 1.

The custom research software streamlined the process of recording many ECAPs at once in several ways. The forward-masking artefact reduction technique consists of four recording frames: (A) masker alone, (B) masker + probe, (C) probe alone, and (D) system signature. Recording all combinations of masker and probe with the recording electrode placed relative to the location of the probe would involve *repeating* the measurements of the (C) and (D) frames for each ECAP. Therefore, these frames were skipped in most cases, and the (C) and (D) frames were only recorded when the probe and the masker were located on the same electrode. The (C) and (D) frames were then re-used for the remainder of that row of the ${M}_{0}$ matrix. This saved a little less than 50% of the recording time that would otherwise have been necessary. Similar approaches in the Cochlear platform have been shown to incorporate minimal error into ECAP amplitude measures [32]. Secondly, the use of a fast USB-to-serial connection converter enabled faster communication between the testing computer and the research hardware (consisting of the Clarion Programming Interface version II and a Platinum Series Processor (PSP)). With these two factors incorporated, the data collection time for the ${M}_{0}$ matrix amounted to approximately 28 min per participant.

Inclusion criteria for the study related to a reliability metric that was calculated between repeat measures of the recorded ${M}_{0}$ for each research participant. Garcia et al*.* showed that the PECAP algorithm can re-produce underlying neural excitation patterns defined as a combination of current spread and neural responsiveness with 90% accuracy down to a signal-to-noise ratio (SNR) of 10 dB [26]. This was shown using computer simulations where the underlying “ground truth” of the neural excitation patterns was pre-defined. Below this SNR, the accuracy of the algorithm to re-create the ground truth dropped below 90%. Therefore, if the ${M}_{0}$ of any of the 15 ears included in this study was below 10 dB, then they were excluded from analysis. The SNR was determined by calculating the root mean squared error (RMSE) between repeated measurements of ECAPs for each participant. Repeated measures were available for the ECAPs recorded where the masker and the probe were presented on the same electrode (the “diagonal” of ${M}_{0}$) as part of the standard PECAP procedure. The SNR could therefore be calculated from this RMSE using a transfer function defined in Garcia et al*.* [26] and shown below in Eq. 1.

1 $$f\left(x\right)= -5.70\times {10}^{-7}{x}^{5}+1.15\times {10}^{-5}{x}^{4}+8.44\times {10}^{-4}{x}^{3}-0.012\;{\times \;x}^{2}-0.80x+17.46$$

As indicated in Table 1, the SNR did not reach the 10 dB threshold for three of the 15 ears, and therefore, data from AB011R, AB018R, and AB020L were excluded from analysis.

The ${M}_{0}$ s for the remaining twelve ears were then submitted to the Panoramic ECAP algorithm described in Eqs. 1–10 in Garcia et al*.* to estimate current spread and neural responsiveness for each electrode switched on in the participants’ respective MAPs [26]. In brief, the algorithm constructs neural activation patterns based on a convolution of neural responsiveness and Gaussian current spread centered at each electrode, and reconstructs the expected $M$ given the estimated neural activation patterns. The current spread and neural responsiveness estimates are then allowed to change within an optimization loop that minimizes the least squared error between the expected $M$ and the measured ${M}_{0}$.

#### Blurring Stimulation Condition

**Fig. 1:**
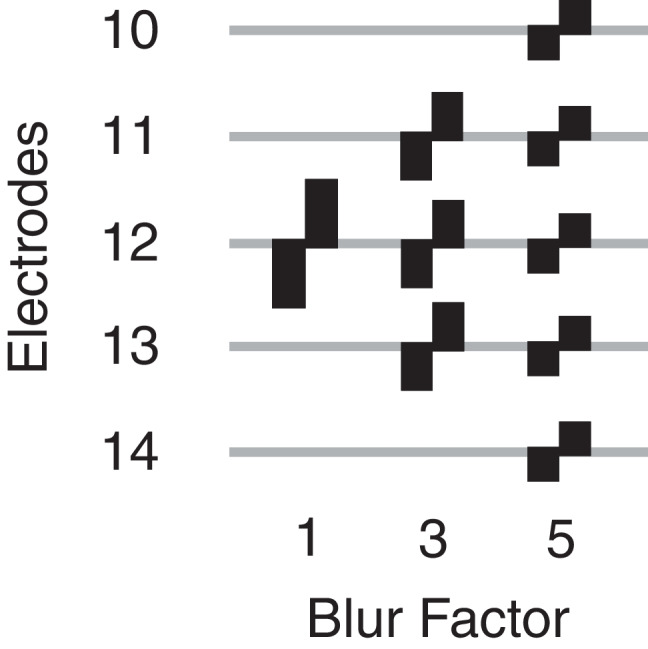
Graphic representing the stimulation configuration when one electrode is stimulated on its own (baseline condition, blur factor = 1), where three adjacent electrodes are stimulated simultaneously (blur factor = 3), and where five adjacent electrodes are stimulated simultaneously (blur factor = 5)

The two electrodes with the highest and lowest focused (sQP) thresholds, respectively (as described above), were selected for each research participant to stimulate with blurred current (see Table 1 for details). There were two blurring factors evaluated: first, one electrode was additionally stimulated on each side of the central stimulating electrode constituting “Blur Factor 3,” and second, two electrodes were additionally stimulated on each side of the central stimulating electrode constituting “Blur Factor 5” (graphically depicted in Fig. 1). Each of the electrodes stimulated simultaneously was presented with the same amount of electrical current. The blur factors of 3 and 5 electrodes were selected based on results from Goehring et al*.* that showed that speech-in-noise recognition thresholds (SRTs) started to increase compared to a stimulation in standard monopolar mode when all channels were blurred by using 4 electrodes simultaneously [21]. In this condition, a stimulating “channel” will refer not directly to a single electrode but to the central stimulating electrode in a group of 3 or 5 simultaneously stimulated electrodes.

The selection of the channels for the blurring conditions was necessarily limited by the study design; in order for an electrode to be the central blurred electrode, there had to be at least 2 electrodes available on each side to additionally stimulate for the “Blur Factor 5” condition and an additional electrode on each side to serve as the recording electrode for the ECAP. Therefore, if all electrodes were active in a CI listener’s MAP, the most apical channel that could be blurred was centered on electrode 4, and the most basal channel that could be blurred was centered on electrode 13. As the original study design also included a condition wherein 7 electrodes would be stimulated simultaneously, the software further restricted this to between channels centered on electrodes 5 and 12. However, due to time constraints for the data collection, it was not possible to measure the 7-electrode configuration.

Loudness scaling was performed for each of the two channels selected for blurred stimulation for both of the blur factors. This was done to determine the current level required for the level rating selected for the monopolar ${M}_{0}$ for that participant for both blur factors (this was not always the default level rating of 6 or MCL; see Table 1). ECAPs were then additionally collected for both these channels and at both blur factor 3 and blur factor 5 for each masker-probe combination condition that involved the central electrode of the channel in question. The blurred stimulation was applied both for the case where the blurring channel was centered on the masker and where it was centered on the probe electrode. This only required additional ECAPs to be recorded in blurring conditions for the row and column in the ${M}_{0}$ matrix that involved the blurred electrodes. This row and column of ECAP data were then inserted into the ${M}_{0}$ matrix obtained for the baseline condition, replacing the monopolar ECAP amplitudes for these electrodes. The resultant $M$ matrices will be referred to as the ${M}_{blur}$ matrices. There were four of these ${M}_{blur}$ matrices in total, consisting of both blur factors (3 vs 5 electrodes) and both sQP threshold levels (high vs low). The four ${M}_{blur}$ matrices were then submitted to the PECAP algorithm to calculate estimates of current spread (*σ*) and neural responsiveness (*η*) for each electrode.

#### Focused Stimulation Condition

The procedure described above was also implemented for partial tripolar (pTP) stimulation. This was conducted for the same two electrodes for each participant as in the blurring stimulation condition, with the exception of AB011L and AB013L for whom the basal electrode for stimulating in pTP mode was shifted 1 electrode more basal compared to with the blurred mode (see Table 1).

**Fig. 2:**
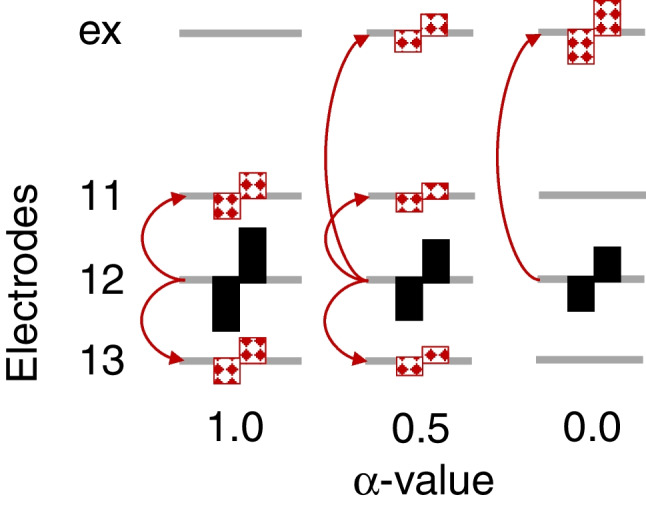
Graphic representing the stimulation configuration when in full tripolar mode where all current is returned to the neighboring electrodes (*α* = 1.0), a partial tripolar mode when 25% of the current is returned to each of the neighboring electrodes and 50% is returned to the extra-cochlear electrode (*α* = 0.5), and monopolar mode when all current is returned to the extra-cochlear electrode (*α* = 0.0). Solid black pulses indicate active current and red, patterned pulses indicate return current. (ex, extra-cochlear electrode)

Partial tripolar mode consists of sending active current to one central electrode and returning part of the current to the two adjacent electrodes and the remainder of the current to an extra-cochlear electrode. For this stimulation, the portion of the return current evenly split between the two adjacent electrodes is referred to as the *α*-value. Therefore, *α* = 1.0 indicates that 50% of the current is returned to the electrode immediately apical to the central active stimulating electrode, and 50% of the current is returned to the electrode immediately basal to it. By contrast, *α* = 0.5 indicates that 25% of the active current is returned to each of the flanking electrodes and 50% of the current is returned to an extra-cochlear electrode (see Fig. 2). For this portion of the experiment, the *α*-value was determined by starting with *α* = 1.0 and increasing the current level until either a loudness level of “8” was achieved or the stimulation compliance limit was reached for that electrode. If the latter was reached first, then the *α*-value was decreased and the procedure repeated until a loudness level of “8” could be achieved. If this was not possible even at *α* = 0.5, then the focused stimulation portion of the study was skipped for this participant. This was the case for 6 of the 15 ears (AB002R, AB014L, AB016L, AB017L, AB018R, and AB020L; see Table 1). The *α*-value was varied in this way to find the highest degree of focusing possible within compliance limits, with the ultimate goal of achieving a current distribution as similar as possible to full tripolar stimulation where no current is returned to extra-cochlear electrodes. The *α*-value for each remaining participant can be found in Table 1.

**Fig. 3:**
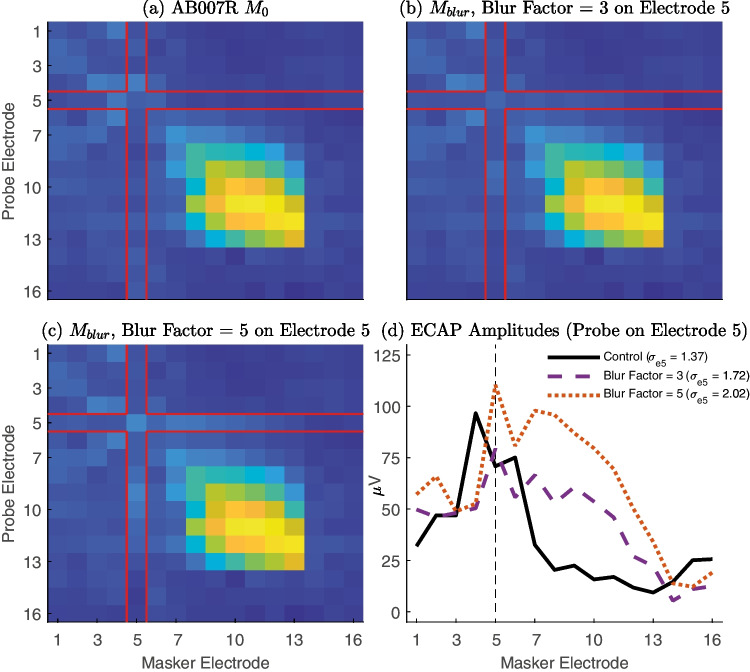
(**a**) ${M}_{0}$ matrix for AB007R, with each cell of the heat map representing the ECAP amplitude in µV of the masker-probe electrode combination indicated by its location. The red lines indicate which cells of the matrix are manipulated in the blur conditions displayed in (b, c). (**b**) ${M}_{blur}$ for the high sQP threshold electrode (5) for blur factor = 3. The red lines represent the cells of the matrix that were stimulated with 3 electrodes simultaneously (electrodes 4–6) when either the masker or the probe was centered on electrode 5. (**c**) ${M}_{blur}$ for the high sQP threshold electrode (5) for blur factor = 5. The red lines represent the cells of the matrix that were stimulated with 5 electrodes simultaneously (electrodes 3–7) when either the masker or the probe was centered on electrode 5. (**d**) ECAP amplitudes for the row of each $M$ matrix in (a)–(c) representing the case where the probe is presented on electrode 5, including PECAP’s current spread estimate for electrode 5. (sQP, steered quadrupolar stimulation; *σ*, PECAP’s current spread estimate; e, electrode)

**Fig. 4:**
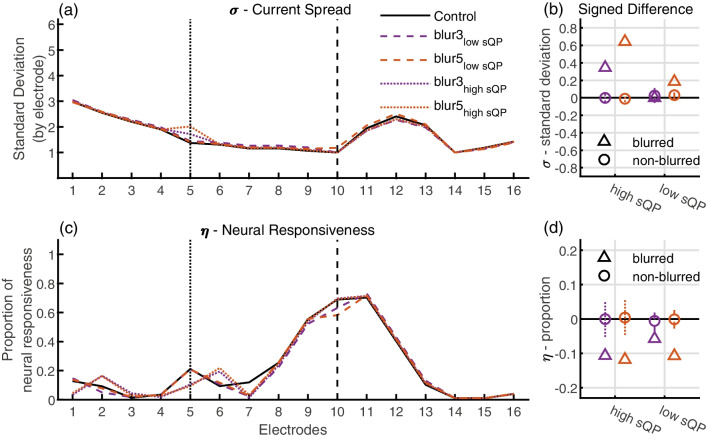
PECAP estimates of current spread (**a**) and neural responsiveness (**c**) for the baseline and blurring conditions for AB007R. The black solid lines represent the baseline condition where no manipulations were made to the spread of electrical current and each electrode was stimulated in standard monopolar mode. The purple lines represent the PECAP results where three electrodes were stimulated simultaneously (blur factor 3), and the orange lines represent the case where five electrodes were stimulated simultaneously (blur factor 5). The vertical black dotted lines represent the electrode that showed the highest focused threshold (sQP), and the vertical black dashed lines represent the electrode that showed the lowest focused threshold; these are the electrodes that were blurred for this participant. The colored dotted lines represent the PECAP results from the scenarios where the electrode on which blurred stimulation was applied showed a high focused threshold, and the colored dashed lines represent where it showed a low focused threshold. Signed differences between the baseline conditions and the manipulated conditions for blurred electrodes (represented by triangles) and for the non-blurred electrodes that were stimulated in standard monopolar mode (represented by circles) are displayed for the current spread estimate (**b**) and for the neural responsiveness estimate (**d**). The error bars represent one standard deviation above and below the mean. (sQP, steered quadrupolar stimulation)

**Fig. 5:**
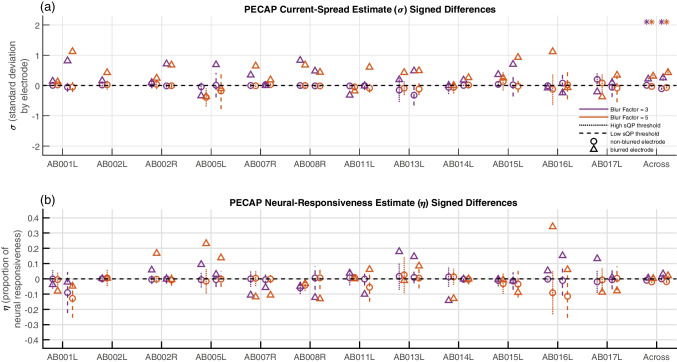
Signed differences for PECAP estimates of current spread (**a**) and neural responsiveness (**b**). The triangles represent the blurred electrodes and the circles represent the non-blurred electrodes. The purple symbols represent blur factor = 3 and the orange represent blur factor = 5. Dotted lines (oriented towards the left for each participant) represent electrodes that showed high sQP thresholds and dashed lines (oriented towards the right for each participant) represent electrodes that showed low sQP thresholds. Error bars represent one standard deviation above and below the mean. Across participants, asterisks (*) are corrected for multiple comparisons within the ANOVA and represent 95% significance within a Tukey–Kramer comparison. (sQP, steered quadrupolar stimulation; ANOVA, analysis of variance; *p < 0.05)

**Fig. 6:**
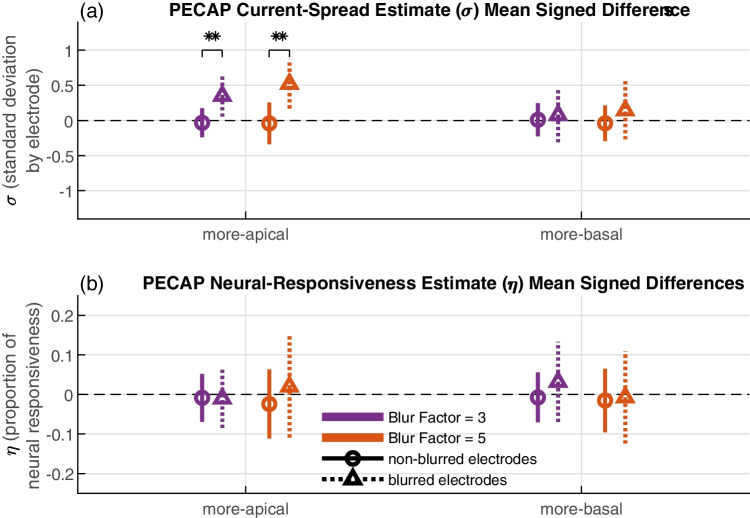
Across-electrode signed differences for PECAP estimates of current spread (**a**) and neural responsiveness (**b**). The triangles and the dotted lines represent the blurred electrodes and the circles and the solid lines represent the non-blurred electrodes. The purple symbols (oriented towards the left of each group) represent the blur factor = 3 and the orange (oriented towards the right of each group) represent blur factor = 5. The three-way ANOVA showed significant main effects of blurring (circles vs triangles, *p* < 0.0001) and of electrode location (more-apical vs more-basal, *p* = 0.0001), and a significant interaction between the two (*p* < 0.0001) on the current spread estimate (top), suggesting that the effect of blurring was effective at the more-apical electrodes but not at the basal electrodes. No significant effects were found in the three-way ANOVA on the neural responsiveness estimate. Error bars represent one standard deviation above and below the mean. (**p < 0.001)

**Fig. 7:**
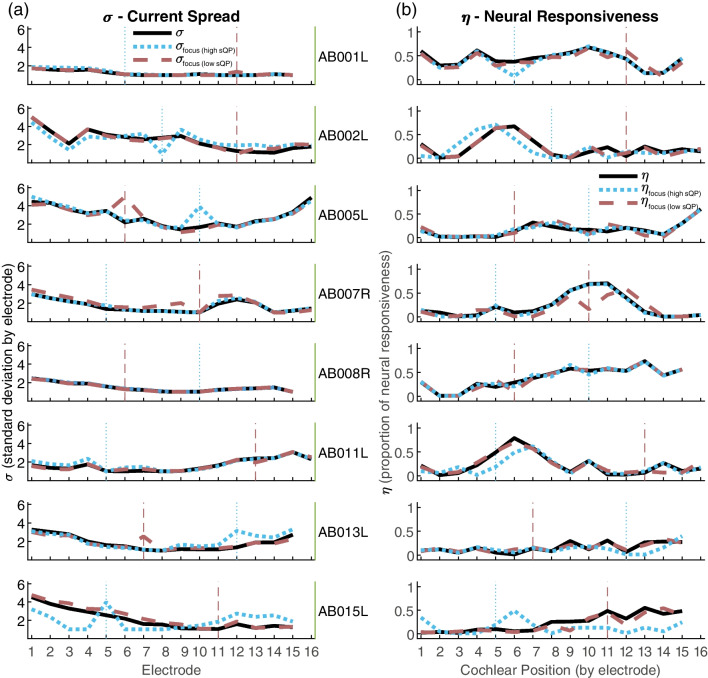
PECAP estimates of current spread (**a**) and neural responsiveness (**b**) for each of the 8 participants retained in the focused stimulation portion of the study. Shown are the baseline condition (solid black lines), the condition where the electrode stimulated in pTP mode showed a high sQP threshold (light-blue dotted lines), and the condition where the electrode stimulated in pTP mode showed a low sQP threshold (gray-pink dashed lines). The vertical light-blue dotted lines indicate the electrode with the highest sQP threshold for that participant and the vertical gray-pink dashed lines indicate the electrode with the lowest sQP threshold for that participant. (sQP, steered quadrupolar)

**Fig. 8:**
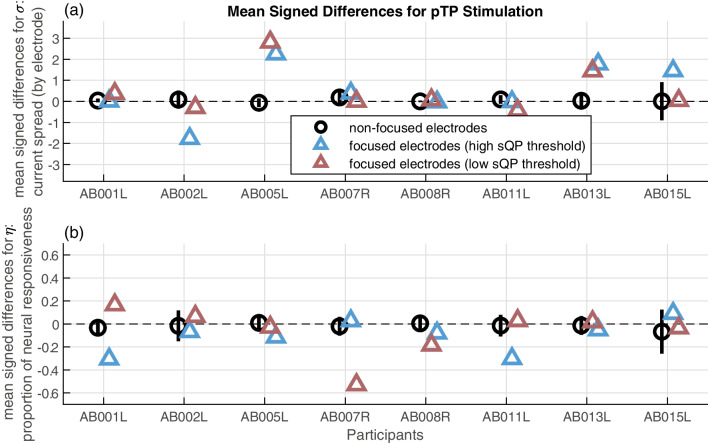
Signed differences for PECAP’s current spread (*σ*) estimate (**a**) and neural responsiveness (*ƞ*) estimate (**b**) for each of the 8 participants retained in the focusing portion of the study. The black circles represent the non-manipulated or non-focused electrodes, and the associated error bars represent one standard deviation above and below the mean. The blue triangles represent the focused electrodes that showed high sQP thresholds, and the pink triangles represent the focused electrodes that showed the low sQP thresholds

As in the case for the blurring stimulation conditions, ECAPs were only recorded for each masker-probe combination that involved the central pTP electrodes in question, making up one row and column in the ${M}_{0}$ matrix. This row and column of ECAP data were then inserted into the ${M}_{0}$ matrix obtained for the baseline condition, replacing the monopolar ECAP amplitudes for these electrodes. The resultant $M$ matrices will be referred to as the ${M}_{pTP}$ matrices. The two ${M}_{pTP}$ matrices were then submitted to the PECAP algorithm to calculate estimates of current spread (*σ*) and neural responsiveness (*η*) for each electrode.

### Hypotheses and Statistical Analysis

#### Planned Hypotheses

First, if the PECAP algorithm is able to detect increases in current spread correctly, then the estimated current spread (*σ*) for the channels on which electrical stimulation was blurred should be higher than in the baseline condition. Likewise, if PECAP is able to attribute the blurred current to the correct channel, then there should be no impact on the current spread estimate for non-blurred channels, and if it is also able to separate its estimates of the two factors characterizing the electrode-neuron interface, then there should not be an effect on the neural responsiveness estimate.

Second, it was hypothesized that simultaneously stimulating five adjacent electrodes (blur factor 5) would produce a greater increase in current spread than simultaneously stimulating three adjacent electrodes (blur factor 3).

Third, we hypothesized that if partial tripolar mode focuses electrical current, then PECAP would predict a decrease in the current spread in the focused condition for the electrodes stimulated in partial tripolar mode compared to the baseline condition. Likewise, if PECAP is able to attribute the focused current to the correct electrode, then there should not be an impact on the current spread estimate for non-focused electrodes, and if PECAP is able to separate its estimates of the two factors characterizing the electrode-neuron interface, then there should not be an effect on the neural responsiveness estimate.

We also investigated whether the effectiveness of both the blurring and the pTP stimulations on PECAP’s current spread estimate differed between electrodes with high vs low focused thresholds. It has been argued that low focused thresholds reflect regions of good neural health [31] and there are ways in which this might moderate the effectiveness of the blurring or pTP manipulations. For example, increasing current spread (blurring) beyond a highly localized region of high neural responsiveness might have a minimal effect on the neural spread of excitation, because the current would spread to a less-responsive region. Alternatively, a broad region of high neural health might mean that MCL can be reached with a narrow current spread, in which case blurring might have a large effect on neural excitation.

### Statistical Analysis

Signed differences were calculated for both PECAP’s current spread (*σ*) and neural responsiveness (*η*) estimates between the baseline conditions and each of the four blurring conditions as well as the two focusing conditions for each research participant. This was done in order to evaluate and quantify the size of the effect for electrical current manipulations on PECAP estimates of the electrode-neuron interface. Instead of directly assessing the current spread (*σ*) and neural responsiveness (*η*) estimates, signed differences in these estimates were chosen as the dependent variables in order to remove between-subject variation representative of the heterogeneity known to be characteristic of most behavioral and electrophysiological assessments of cochlear implant perception. The signed differences were separated into “manipulated” electrodes — representing the central electrodes that were presented with either blurred or partial tripolar stimulation and listed in Table 1 — and “non-manipulated” electrodes — representing the remainder of the electrode array stimulated in standard monopolar mode. The manipulated group contained 1 data point per condition per participant by design, whereas the non-manipulated group contained either 15 or 14 values, depending on how many electrodes were switched on in the participant’s MAP and therefore included in the PECAP measurements. The individual values within the non-manipulated group are each used in the analysis instead of calculating their means in order to retain information about their variance. A positive signed difference would indicate that the effect of current manipulation *increased* the spread of electrical current (or neural responsiveness) for the manipulated condition compared to the baseline condition. All analyses were done using MATLAB version 2020b (Mathworks, Natick, MA, USA).

For the blurring conditions, two 3-way analyses of variance (ANOVAs) were conducted, one for the current spread and another for the neural responsiveness estimates. Three factors each with two levels were submitted to both ANOVAs: Factor 1: blurring (blurred vs non-blurred electrodes), Factor 2: blur factor (Blur Factor 3 vs Blur Factor 5), and Factor 3: sQP threshold (high vs low). This was done to determine if there was an effect of blurring overall, if there was a difference between the two blurring factors, and whether there was an effect of sQP threshold on the blurring effect, respectively. Residuals were checked for normality prior to reporting results.

For the focusing conditions, two 2-way ANOVAs were conducted, one for the current spread and another for the neural responsiveness estimates. Two factors each with two levels were submitted to both ANOVAs: Factor 1: focusing (focused vs non-focused electrodes), and Factor 2: sQP threshold (high vs low). Residuals were checked for normality prior to reporting results.

A few post hoc statistical tests were also performed and are described in the “Results” section. Note that the statistical tests performed evaluated the presence of effects, but could not confirm their absence.

## Results

### Blurring Condition

The ${M}_{0}$ and ${M}_{blur}$ matrices were submitted to the PECAP algorithm and estimates of current spread (*σ*) and neural responsiveness (*ƞ*) were extracted for each participant and for each condition. For one example participant (AB007R), ${M}_{0}$ and ${M}_{blur}$ matrices for the low sQP electrode (5) for both blur factor conditions are shown in Fig. 3a, b and c, respectively. Figure 3d shows ECAP amplitudes for the row of each $M$ where the probe stimulation is centered electrode 5. Signed differences were calculated for both estimates between the baseline conditions (${\sigma }_{0}$ and ${\eta }_{0}$) and the blurred conditions (${\sigma }_{blur}$ and ${\eta }_{blur}$) for both the electrodes on which blurred stimulation was applied (hereafter referred to as the “blurred electrodes”) and the electrodes on which the blurred stimulation was not applied (hereafter referred to as the “non-blurred electrodes”). The PECAP estimates (a and c) and the signed differences (b and d) from AB007R are displayed in Fig. 4. Note that the signed differences for the non-manipulated electrodes (circles, Fig. 4b and d) are all close to zero for this participant, consistent with our expectation that blurring one electrode should not affect the current spread or neural responsiveness estimates for the other non-blurred electrodes.

The signed differences of the current spread (*σ*) estimate were then submitted to a three-way ANOVA to investigate the effect of (1) blurred stimulation (blurred vs non-blurred electrodes), (2) blur factor (3 vs 5 adjacent electrodes simultaneously stimulated), and (3) focused threshold (high vs low sQP threshold) on PECAP’s current spread estimate. The ANOVA showed a significant difference between blurred and non-blurred electrodes (*F*(1,705) = 50.94, *p* < 0.0001), but did not show significant effects of focused threshold (*F*(1,705) = 0, *p* = 0.9524) nor blur factor (*F*(1,705) = 1.42, *p* = 0.2344). The signed differences of the neural responsiveness (*ƞ*) estimate were then submitted to an ANOVA with the same three factors which did not show a significant effect of blurred stimulation (*F*(1,705) = 1.41, *p* = 0.2363), focused threshold (*F*(1,705) = 0.38, *p* = 0.5388), nor blur factor (*F*(1,705) = 0.06, *p* = 0.8053). The signed differences for each individual participant are shown for the current spread estimate in Fig. 5a and for the neural responsiveness estimate in Fig. 5b. In this figure, triangles represent the manipulated (blurred) electrodes, the circles represent the non-manipulated (non-blurred) electrodes, and a positive signed difference indicates that the current spread or neural responsiveness estimate was higher for the manipulated condition than for the baseline condition. As can be observed in the current spread estimate graphs, the confidence intervals for the circles often cross the origin, whereas the triangles are often positive. These cases show situations where the effect of blurring was found in the expected direction.

An additional post hoc ANOVA was computed to investigate whether there was an effect on PECAP’s estimate of current spread of applying blurred stimulation to electrodes towards the apical side of the array compared to towards the basal side of the array. The electrodes towards the apex were at an average of electrode 5.75 ± 0.97 electrodes, and the electrodes towards the base were 10.55 ± 0.93 (where the number following the ± indicates one standard deviation from the mean). Given the non-significant *p*-value of the focused threshold factor in the original ANOVA, this post hoc three-way ANOVA was calculated by replacing the focused threshold factor with the electrode location (more-apical vs more-basal side). This ANOVA again showed a significant effect of blurred stimulation (*F*(1,705) = 57.8, *p* < 0.0001). Interestingly, it also showed a significant effect of electrode location (*F*(1,705) = 14.81, *p* = 0.0001), and a significant interaction between the two main effects (*F*(1,705) = 19.57, *p* < 0.0001). There again was no significant effect of blur factor (*F*(1,705) = 1.32, *p* = 0.2512). These results, summarized in Fig. 6a, show that blurring had a significant effect for the more-apical electrodes that were blurred, and that this effect was significantly larger than for the more-basal electrode. The same ANOVA was repeated with PECAP’s neural responsiveness estimate as the dependent variable; this revealed no significant effect of blurred stimulation (*F*(1,705) = 3.75, *p* = 0.0531), electrode location (*F*(1,705) = 0.28, *p* = 0.5946), nor blur factor (*F*(1,705) = 0.41, *p* = 0.5240) and is displayed in Fig. 6b.

In these 3-way ANOVAs, six separate statistical tests were performed (3 main effects and 3 two-way interactions), requiring a correction for multiple (six) comparisons [33]. Given that four ANOVAs were calculated in total (containing either the “sQP threshold” and “electrode location” for both PECAP’s estimate of current spread and neural responsiveness), one could additionally argue the need for correcting for a further 4 comparisons. All reported significant interactions survive corrections for ten comparisons using the Bonferroni-Dunn method (6 comparisons: *α* = 0.00833, 10 comparisons: *α* = 0.005) [34]. However, it should be noted that a result of observing *no* effect of blurred stimulation on the neural responsiveness estimate would provide a lack of evidence against PECAP’s ability to attribute manipulations of current to the correct estimate. Therefore, applying this correction could inflate the risk of type II errors (false negatives). The ANOVA for the neural responsiveness estimate revealed non-significant *p*-values for the blurred stimulation factor even with the uncorrected case (*p* = 0.2363; 0.0531), but for the post hoc ANOVA this was very close to the significance threshold of *α* = 0.05. Because the output of the PECAP algorithm is slightly affected by the random initial values of the *σ* and *ƞ* vectors that are initiated from random values, a further post hoc analysis was conducted to determine the *p*-value *distribution* for this factor if the PECAP analysis was run 1000 times. Bootstrapping was then conducted, sampling with replacement from these 1000 analyses, to determine confidence intervals for the 95% upper confidence limits (UCL) of the *p*-values for this factor. Note that for a completely random factor, by definition the *p*-value distribution would be flat, with 5% of values falling below the 95% confidence (*α* = 0.05) threshold, by definition.

While the peak of the *p*-value distribution for the effect of blurred stimulation on PECAP’s neural responsiveness estimate was at 0.007 (just below *α* = 0.0083), the UCL was between *p* = 0.18–0.27 (95% confidence intervals). This suggests that the data do not show 95% confidence that the *p*-value would fall below even the uncorrected *α* threshold of 0.05. Furthermore, this particular comparison has a very high power even with a small sample size (*n* = 12, *F* = 3.75, Power > 99%) due to the within-subject study design, as calculated in G*Power version 3.1.9.2 (Universität Kiel, Germany). In terms of frequentist statistics, it is actually *less* likely for an effect to be significant in the long run (i.e., if the study is repeated multiple times) when a single study with high power shows a marginal *p*-value (i.e., between 0.02 and 0.05) as shown by Lakens [35, 36]. Given this and the high power of the statistical test, it is unlikely that a significant effect of blurring on the neural responsiveness estimate would be found if the study were to be repeated.

Following the finding of the significant effect of electrode location on blurring, an additional post hoc 2-way ANOVA was performed to assess the effect of blur factor (3 vs 5 electrodes) and location (more-apical vs more-basal) on the difference in current level required for MCL when compared to the baseline condition where only one electrode was stimulated at any given time (i.e., blur factor 1). This ANOVA showed no significant main effect for the electrode location (*F*(1,44) = 0, *p* = 0.9802), nor of blur factor (*F*(1,44) = 3.87, *p* = 0.0556), nor did it show a significant effect for the interaction between the two main effects (*F*(1,44) = 0, *p* = 0.9836) on the current level required for MCL compared to the baseline condition.

### Focusing Condition

The ${M}_{0}$ and ${M}_{pTP}$ matrices were submitted to the PECAP algorithm and estimates of current spread (*σ*) and neural responsiveness (*ƞ*) were extracted for each participant and for each condition. The current spread and neural responsiveness results are displayed in Fig. 7a and b, respectively.

The signed differences of the current spread (*σ*) estimate were then submitted to a two-way ANOVA to investigate the effect of (1) pTP stimulation and (2) focused threshold on PECAP’s current spread estimate. The ANOVA showed a significant effect of pTP stimulation (*F*(1,244) = 6.87, *p* = 0.0093), but did not show a significant effect of focused threshold (*F*(1,244) = 0.48, *p* = 0.6193). The signed differences of the neural responsiveness (*ƞ*) estimate were then submitted to the same two-way ANOVA that did not show a significant effect of pTP stimulation (*F*(1,244) = 2.84, *p* = 0.0934) nor of focused threshold (*F*(1,244) = 0.01, *p* = 0.9939). To mimic the post hoc analysis done for the blurring condition, the focused threshold factor was replaced with the electrode location factor in two post hoc two-way ANOVAs, neither of which found significant effects of electrode location (*σ*: *F*(1,244) = 0.02, *p* = 0.8948; *ƞ*: *F*(1,244) = 0.27, *p* = 0.6023).

The direction of the effect of focusing on PECAP’s current spread estimate was in the opposite direction of that predicted: at the group level, stimulating in partial tripolar mode appeared to *increase* the current spread. However, this effect seemed to be driven primarily by 2 of the 8 participants. The signed differences for each individual participant are displayed for the current spread and neural responsiveness estimates in Fig. 8a and b, respectively. It can be observed that for AB005L and AB013L, as well as one electrode for AB015L, PECAP appears to demonstrate an increase in current spread with pTP stimulation compared to the MP condition. However, for one of the electrodes in AB002L, PECAP appears to predict decreased current spread for the electrode stimulating in pTP mode, and for the remainder of the participants, stimulating in pTP mode appears to have had no significant effect on the current spread compared to MP mode.

## Discussion

The significant effect of blurring on PECAP’s current spread estimate across participants suggests that PECAP is capable of detecting localized manipulations of current spread when multiple adjacent electrodes are stimulated simultaneously. This provides evidence in support of the accuracy of PECAP’s current spread estimate. The size of the across-participant effect was relatively small; however, at about 0.5 *σ*. PECAP’s estimate of current spread (*σ*) describes the width of a Gaussian curve and is measured in units of electrodes, reflecting an across-participant effect of (≈ 0.5 × 2) ≈ 1 electrode’s-worth of additional current spread caused by stimulating with either 3 or 5 adjacent electrodes simultaneously in comparison to using a single electrode for stimulation. In the ideal but unrealistic scenario where no overlap exists between adjacent electrodes’ excitation patterns in standard monopolar mode, one might have expected that stimulating with 3 or 5 electrodes simultaneously might cause 2 or 4 electrode’s-worth of additional current spread (analogous to an effect on *σ* of 1 or 2). The observed effect is much smaller than this because there is considerable overlap in the neural populations stimulated by adjacent electrodes in monopolar mode. The overlap between electrodes is a well-known limitation of CIs and methods such as PECAP are required for objective measurement.

Although we found a significant effect of blurring on PECAP’s estimate of current spread, there was no significant difference found between the two Blur Factors (3 vs 5 electrodes) assessed. This may either be because no effect of Blur Factor exists, or because the PECAP algorithm was unable to detect an effect of Blur Factor. These results do align with what was found by Goehring et al*.* in their study of the effects of blurring on speech reception thresholds. They simultaneously stimulated 2, 3, 4, or 6 adjacent electrodes and while statistically significant differences of 3–4 dB were found between SRTs with 1 electrode compared to 4 or 6 electrodes, no differences were found between 3 electrodes and 4 or 6 electrodes [21]. As they *did* report a significant difference of 4.1 dB between 6 and 8 simultaneously stimulated electrodes, we expect that future work may likely demonstrate that PECAP would be sensitive to the Blur Factor if a condition is assessed where more than 5 electrodes are simultaneously stimulated.

The blurring manipulation did not produce a significant effect on PECAP’s estimate of neural responsiveness (*p* = 0.053), and the high power (> 99%) of the statistical test in this case suggests that we would be unlikely to find a significant effect if the experiment were repeated. Nevertheless, it may be beneficial to investigate alterations to the PECAP algorithm to determine whether it is possible to more effectively separate the current spread and neural responsiveness estimates. Alternative versions of the PECAP algorithm have been proposed and investigated extensively in computer simulations, but not assessed using a study design where current was manipulated in human CI users [27]. It is possible that one of the limitations of PECAP is its modelling of current spread using symmetric Gaussian curves, as cochlear anatomy of wider Scala diameters towards the base compared to the apex [37, 38] suggests that the spread of electrical current within the cochlea may not be symmetric along the apical-basal dimension. There is also evidence to suggest that, at least at the level of the electrode array, voltage profiles show a pattern often referred to as “basal shunting” where Stimulation-Current-Induced Non-Stimulating Electrode Voltage recordings (SCINSEVs) [39] show steeper decay of voltage in the basal direction than the apical one [13, 40, 41]. (SCINSEVs are commonly referred to as trans-impedance matrices (TIMs), electrical field imaging (EFIs), or voltage matrices (VM), depending on the device manufacturer, but all involve measuring the voltage on non-stimulated electrodes and scaling them by the input current.) One might then hypothesize that PECAP could better separate the contributions of current spread and neural responsiveness to the neural excitation patterns if it allowed for asymmetric current spread and potentially more accurately model what really occurs in the cochlea. However, blurring the stimulation current had an even greater effect on the neural responsiveness (*ƞ*) estimate when these data were re-analyzed using alternative versions of PECAP described by Garcia (2022) and in which asymmetric current spread was assumed. This suggested that the algorithm assessed in this article and described by Garcia et al. [26] that uses *symmetric* Gaussians to model current spread is better at separating the current spread and neural responsiveness attributes of the electrode-neuron interface than a version that uses asymmetric Gaussians. One reason for this may be that PECAP estimates current spread at the level of the neurons rather than at the electrodes, and while SCINSEVs suggest asymmetric voltage decay in the apical-basal directions at the level of the electrodes, this asymmetry may be marginal at the level of neural activation. Psychophysical measures of spread of excitation — such as masked excitation patterns and psychophysical tuning curves — do not consistently indicate that excitation patterns are steeper on the basal than on the apical edge [19, 42].

It has been suggested previously that focused thresholds represent some aspects of neural health [26, 31], and that they also predict for areas of poor vs good spatial selectivity [29]. Therefore, one might expect the focused thresholds to modulate the effectiveness of the blurred or pTP stimulation. However, we did not find a significant effect of high vs low focused threshold on the effects of either current manipulation between electrodes. A possible explanation for this may be that for some participants, the differences in the focused thresholds across the electrode array were not great enough to modulate the effects of blurring or pTP stimulation on the spread of current.

One interesting and unexpected result from the blurring experiment was the significant post hoc interaction between blurring and location, indicating that increased current spread was detected as a result of blurring towards the apex (average electrode 5.75 ± 0.97) but not towards the base (average electrode 10.55 ± 0.93). Again, due to the study design, the effect of blurring and the detection of the increased current spread caused by the blurring are two confounded factors. This means that we do not know from the analysis alone whether this result suggests that we simply were unable to blur as effectively at the base compared to the apex, or whether PECAP is less sensitive to detecting manipulations to the current spread at the base than at the apex. However, there is no computational consideration within the PECAP algorithm for estimating either neural responsiveness or current spread *differently* at different positions along the implant array. Neither are there differences in electrode spacing at different points along the implant array in the Advanced Bionics platform that could have contributed to the effect. This suggests that it is more likely in this particular case that the effect of blurring is less effective on the basal side of the array than on the apical side. One explanation could be that CI listeners have higher tolerance for loudness of novel (i.e., blurred) stimuli towards the apex, and therefore, the stimulation current levels of the blurred stimuli may simply have been higher relative to the baseline condition than towards the base. However, no effect of cochlear location was found on the difference in current level required to achieve MCL compared to the baseline condition. There may still have been differences in widths of the voltage profiles delivered to the electrodes, as this is also affected by the impedances of the electrodes. However, as SCINSEV measurements were not available in these participants, we were not able to assess this.

Another explanation for the greater effectiveness of blurring towards the apex could be differences in cochlear anatomy. The diameter of the Scala Tympani where the electrodes are inserted is wider at the basal end of the array than the apex, leaving more space for the electrodes to be farther away from the neural tissue at the basal end of the array [7, 37, 38]. This could mean, on average, that current already has a broader spread of excitation at MCL at the basal end of the array, therefore making it more difficult to blur the current compared to the non-blurred condition that it might be at the apex. These data are therefore consistent with the idea that it is easier to blur current in areas of the cochlea where the current is not already blurred due to the cochlear anatomy (i.e., the apex). This is in keeping with the findings of Goehring et al*.* who found that the number of channels of blurring required to reduce performance on speech scores was negatively correlated with performance on a spectro-temporal test; less blurring was required to reduce CI listeners speech perception performance in those that had better overall spectro-temporal resolution [21]. It is also consistent with Goehring et al*.*’s further finding that blurring 5 of 15 apical channels reduced performance on speech tests whereas blurring the same number of middle or basal channels showed no such effect [22]. The authors suggested that this may be due to the greater importance of the spectral information delivered to the apical electrodes for speech intelligibility compared to that delivered to the basal electrodes. However, these new findings suggest that it may also — or alternatively — be because blurring is more effective at the level of the electrode-neuron interface on the apical side of the array than on the basal one. Of course, as this effect was found as the result of a post hoc analysis and not a pre-planned one, future studies should also be done to confirm that this effect is repeatable and robust.

The results of the focusing experiment appear to show variable effects of partial tripolar (pTP) stimulation on PECAP’s estimation of current spread within the cochlea between cochlear implant users. For one electrode for one participant, the expected effect appeared to be observed where PECAP showed narrower current spread for the electrode on which pTP stimulation was used compared to the baseline condition. For four out of eight participants, there did not appear to be an overall effect of stimulating in pTP mode on PECAP’s estimate of current spread compared to MP mode. However, for two participants, both electrodes stimulated in pTP mode appeared to show *increases* in current spread compared to the baseline condition. These two participants appear to be driving the group-level effect of a moderate increase in current spread as a result of pTP stimulation. 

As with any current-focusing paradigm, while the pTP method of delivering current does narrow the field of electrical stimulation, it also requires more current overall in order to achieve the same loudness level (i.e., MCL) as the standard monopolar stimulation [17]. An increase in overall current applied has the opposite effect on spread of electrical current as the focusing technique. It may be that for the electrodes that appeared to show an increase in current spread as a result of pTP stimulation, the effect of the higher current level required to achieve MCL on broadening the current spread was stronger than the effect of stimulating in pTP mode on narrowing the current spread. This has previously been investigated by Litvak et al*.* who showed this kind of combined effect when comparing relative contributions of the two opposing effects in a computational model [43]. We hypothesize that these two opposing effects are at play for each of the eight participants evaluated in this study, and indeed perhaps in any current-focusing paradigm. However, the relative strength of each of the effects likely affected the overall outcome of whether the current spread appeared reduced or not. This may be a contributing factor to mixed result when employing focused stimulation in CI processing strategies on performance on speech tests and other spectral discrimination tests [14–19].

Another explanation for the apparent lack of effect present in four of the participants might be that the PECAP algorithm could be less sensitive to decreases in current spread than increases. The estimation of current spread within the algorithm depends largely on the amplitude of ECAP’s that represent masker-probe pairs off the diagonal of the ${M}_{0}$ matrix. These are lower in amplitude than the ECAPs on the diagonal (when the masker and probe are presented on the same electrode) and would be reduced even more in the case where the current is successfully focused. In contrast, these off-diagonal ECAPs would increase in amplitude when the current is successfully blurred. In the former case, an effective focusing technique effectively reduces the SNR of the off-diagonal cells in the ${M}_{0}$ matrix. This could contribute to a decrease in sensitivity of the PECAP method and could be an explanation for no apparent effect being observed, especially in cases that already show SNRs in the baseline condition. However, the SNR is above 16 dB for all of the participants in which there did not appear to be effects (see Table 1), so while we cannot rule out that this may have contributed to our results, it seems unlikely to be a key contributing factor.

Other current-focusing techniques may have greater success at narrowing current spread than the partial tripolar stimulation investigated here. Garcia showed that in two out of three participants who participated in a pilot study similar to the one presented in this article, stimulating bipolar mode (BP-0) wherein the active current is returned to one adjacent intra-cochlear electrode revealed narrower current spread predicted by PECAP than when stimulating in monopolar mode (the third participant showed no effect) [27]. Other studies have also shown that stimulating in tripolar mode demonstrates narrower spread of excitation curves compared to monopolar mode, particularly at the level of the inferior colliculus [9, 11, 44, 45]. Other techniques for focusing current have also been investigated, notably the phased-array technique wherein the active current is returned to all other intra-cochlear electrodes, and impedance measures are used to calculate the relative current necessary to be returned to each individual electrode in order to best focus the current profile at the active stimulating electrode [6, 7, 46]. One of the limitations of this technique is that while the desired current profile can be achieved, this is calculated at the level of the electrodes and not at the level of the neurons. Therefore, the current profile presented across the electrode-neuron interface may not reflect the one optimized by the phased-array technique. Indeed, a psychophysical experiment revealed no consistent difference in spread of excitation between phased-array and monopolar stimulation [47], and comparisons between phased-array and TP stimulation fail to reveal more selective stimulation for the former [48]. Another nuanced technique referred to as “Hilltop” has also been investigated wherein a ladder network [49] extended from Vanpoucke et al*.*’s model [40] is used to model voltage profiles using SCINSEV measurements, and this is in turn used to determine how much current to return to each intra-cochlear electrode to create the desired current profile delivered to the electrodes with varying degrees of sharpness applied to the current profile [50]. However, most of these focusing techniques require independent current sources for each electrode contact, and therefore are not possible to employ in all three major CI manufacturer’s hardware.

Another important point is that the PECAP method leverages ECAP measurements that are measured at MCL, and also did so both for the blurred and focused stimulation employed in this study. While the relative contribution of the opposing effect of widening the current profile with increased current required to achieve MCL seemed to outweigh the effect of narrowing the current profile with pTP stimulation for some of the participants described here, this may not be the case at threshold levels or at other points within the dynamic range. Therefore, it is possible that pTP stimulation may be more effective at narrowing current spread than shown here at lower levels in CI user’s dynamic range. As a range of stimulation levels are employed within a clinical coding strategy, it is possible that pTP stimulation may still improve speech perception of spatial selectivity in scenarios that reflect a CI user’s entire dynamic range. This has been suggested before: Carlyon et al*.* point out that Bierer and Faulkner’s observation of sharper patterns for TP than for MP probes when measuring psychophysical tuning curves (PTCs) [29] occurred in a situation where the masker excitation at the neurons stimulated by the probe electrode is quite low [42], and indeed, when recording PTCs the probe is stimulated at a low level in the dynamic range (i.e., 30% above threshold). Methods other than PECAP that can investigate current spread profiles at levels lower than MCL would be useful in investigating these scenarios.

## Conclusion

These experiments provide evidence in favor of the ability of the Panoramic ECAP algorithm to identify localized manipulations of current spread caused by blurred stimulation, and separate its estimates of current spread from its estimate of neural responsiveness. There was no evidence that the effect of blurring was moderated by whether the targeted electrode showed higher or lower focused thresholds. However, only blurred stimulation towards the apical side of the cochlea had an effect of increasing current spread whereas no significant effect was found towards the basal side, consistent with the effect of blurring on speech perception in noise reported by Goehring et al*.* [22]. We found mixed results for the effect of focused current by stimulating in partial tripolar mode on PECAP’s estimate of current spread, suggesting that whereas in some cases the current profile may be narrower compared to stimulation in monopolar mode, in some cases it may have no effect, and in other cases the current profile may widen. This is possibly due to the opposing effect of greater stimulation current level required to reach MCL in partial tripolar mode, and is consistent with modelling work by Litvak et al*.* [43].

The results presented here suggest that the Panoramic ECAP method is an effective tool for characterizing variability in current spread along the length of individual cochlear implant users’ cochleae. They also characterize the effect on current spread of a current manipulation intended to focus current (partial tripolar mode) and one intended to broaden current (blurring by stimulating multiple adjacent electrodes simultaneously). Both of these findings have implications for design of cochlear implant stimulation strategies with an aim to optimize spatial selectivity and ultimately improve speech perception for cochlear implant users.

## Data Availability

Data will be made available upon request of the corresponding author. Custom software developed for data collection can be found here: https://github.com/charlottemgarcia/BlurCAP_and_pTPECAP.
